# Perioperative hyponatremia in total joint arthroplasty: A systematic review of prevalence, risk factors, outcomes, and management

**DOI:** 10.1016/j.jor.2026.05.011

**Published:** 2026-05-16

**Authors:** Abhav Garde, Shlok Patel, Luke Molitor, Joshua R. Porto, Monish S. Lavu, Atul F. Kamath

**Affiliations:** a Case Western Reserve University School of Medicine, Cleveland, OH, 44106, USA; b OrthoNJ, Manasquan, NJ, 08736, USA; c Hackensack Meridian Health, Hackensack, NJ, 07601, USA

**Keywords:** Hyponatremia, Total joint arthroplasty, Total knee arthroplasty, Total hip arthroplasty, Perioperative complication, Sodium optimization

## Abstract

**Background:**

Hyponatremia is the most common electrolyte abnormality in hospitalized patients and frequently occurs in the perioperative period of total joint arthroplasty. Although associated with adverse outcomes, its prevalence, predictors, and optimal management strategies in this population remain incompletely defined. This systematic review evaluated the prevalence of perioperative hyponatremia, identified predictors of postoperative hyponatremia, and assessed associated outcomes and management strategies in primary total hip and knee arthroplasty.

**Methods:**

PubMed, Google Scholar, and EBSCOhost were searched from January 2000 through December 2023. Studies evaluating prevalence, risk factors, outcomes, or management of perioperative hyponatremia in primary total hip or knee arthroplasty were included. Revision and non-primary procedures were excluded. Twenty studies met inclusion criteria. Risk of bias was evaluated via the Methodological Index for Nonrandomized Studies tool, and the mean score was 19.7 ± 1.4. Due to heterogeneity, results were synthesized qualitatively.

**Results:**

Preoperative hyponatremia ranged from 2.1% to 17.8%, whereas postoperative rates ranged from 2.3% to 41.3%, with most cases developing within 24 to 48 h after surgery. Abnormal preoperative sodium was the strongest predictor of postoperative hyponatremia (odds ratios up to 12.33). Preoperative hyponatremia was consistently associated with increased length of stay, readmission, reoperation, periprosthetic fracture, delirium, and medical complications. Limited evidence suggests preoperative sodium optimization may reduce postoperative hyponatremia, whereas correction prior to discharge did not consistently improve outcomes.

**Conclusion:**

Perioperative hyponatremia is common in total joint arthroplasty and is associated with worse outcomes, particularly when present preoperatively. Targeted preoperative screening and optimization may improve perioperative risk stratification and outcomes.

## 1 Introduction

Hyponatremia, commonly defined as serum sodium <135 mmol/L, is the most frequent electrolyte abnormality among hospitalized patients and commonly occurs in the perioperative period of total joint arthroplasty (TJA) 1, 2, 3. Large database studies report preoperative hyponatremia rates around 6.9% in TJA, although individual studies have reported substantially higher rates 4, 5, 6. Perioperative hyponatremia has been associated with worse outcomes after TJA, including greater mortality and length of stay (LOS).^7^^,^^8^ Despite recognition of hyponatremia as a modifiable risk factor in TJA, the optimal approach to perioperative screening and management remains unclear.

The recent shift toward outpatient surgery has complicated routine electrolyte monitoring, emphasizing the need for evidence-based strategies to optimize clinical workflow for screening and treatment of perioperative hyponatremia.^9^ Additionally, many studies found routine postoperative lab testing offers little clinical benefit to the majority of TJA patients and provides associated cost savings 10, 11, 12, 13. Although perioperative hyponatremia is recognized as a clinical concern, few studies have comprehensively synthesized the literature across three key domains: prediction, complications, and management. Similarly, a gap exists in the literature characterizing the relationship between perioperative changes in serum sodium and their associated clinical outcomes.^14^

Thus, an integrated approach systematically analyzing the impact of both preoperative and postoperative hyponatremia in TJA patients is warranted. Specifically, we investigated: (1) What are the predictors for postoperative hyponatremia? (2) What are the implications and associated outcomes of perioperative hyponatremia? (3) What is the efficacy of intervention and management of perioperative hyponatremia?

## 2 Methods

### 2.1 Search strategy and data sources

This systematic review is exempt from Institutional Review Board evaluation due to exclusive utilization of publicly available data lacking personal health identifiers. The study protocol was registered with PROSPERO on January 28th, 2024 (CRD42024503307) and conducted in accordance with Preferred Reporting Items for Systematic Reviews and Meta-Analyses (PRISMA) recommendations.^15^ On December 26th, 2023, we queried the PubMed, Google Scholar, and EBSCOhost electronic databases for studies published between January 1st, 2000 to December 26th, 2023 reporting on prevalence, risk factors, outcomes, and management of perioperative hyponatremia in total joint arthroplasty patients. The full search term used in this study is reported in Appendix 1.

### 2.2 Eligibility criteria

Inclusion criteria required that each study: (1) investigated the prevalence, risk factors, outcomes, and management of perioperative hyponatremia in total joint arthroplasty patients and (2) was available as a full-text English manuscript. Study exclusion criteria were as follows: 1) non-primary arthroplasty procedures (e.g., hemiarthroplasty, unicompartmental arthroplasty, revision arthroplasty); 2) case reports; 3) non-English literature; 4) editorials and commentaries; 5) systematic reviews; 6) posters and abstracts; and 7) duplicate studies.

### 2.3 Study selection

**Fig. 1:**
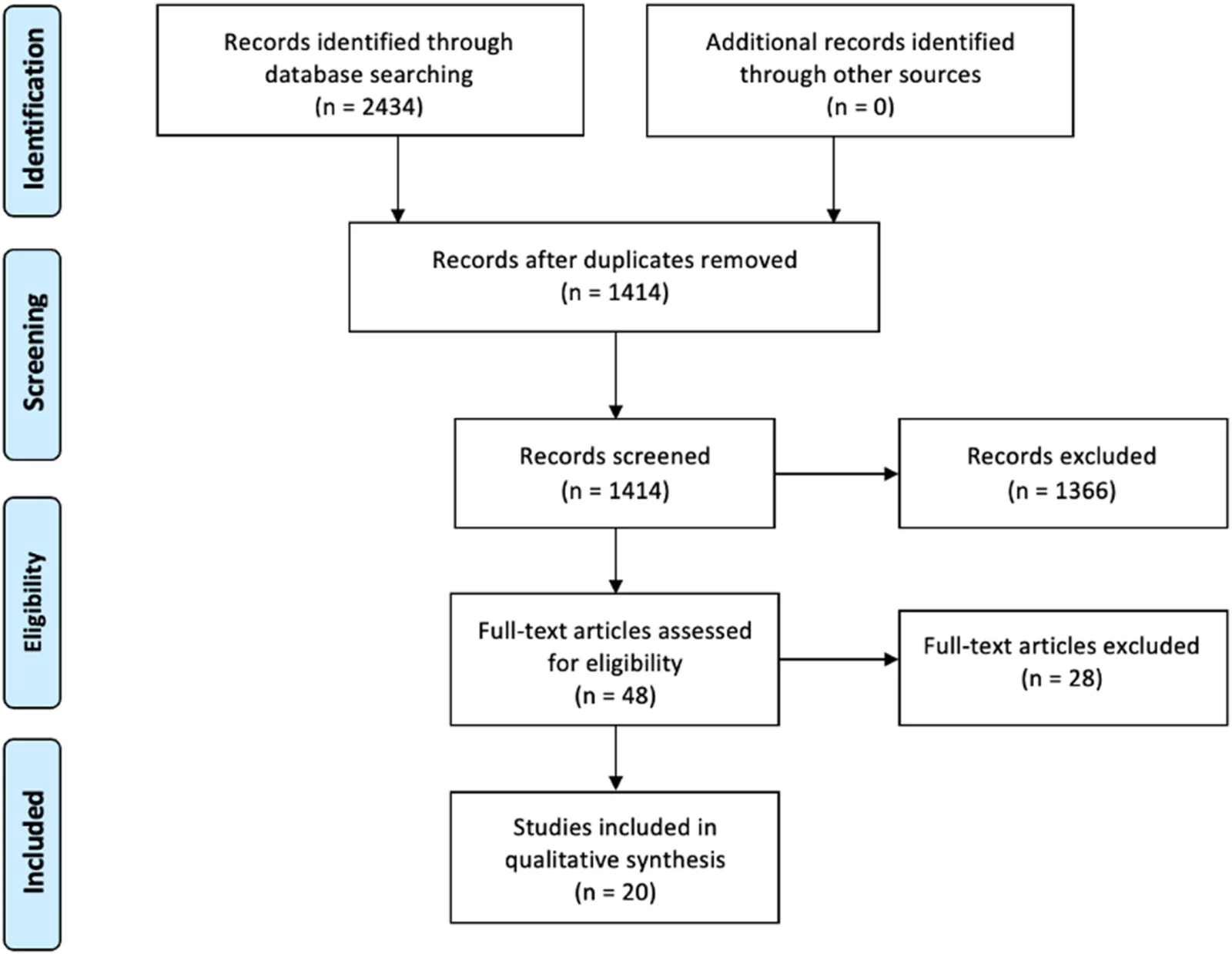
PRISMA diagram depicting the study selection process.

Two independent reviewers (AG and SP) assessed each of the studies for eligibility. To reach consensus, disagreements were discussed with a third reviewer (JP). After duplicates were removed, our search produced 1414 distinct articles, which were then screened for pertinence to our study goals. Following the processes of title and abstract screening, 44 studies were selected for analysis of full text prior to final selection. Of these 44 studies, 20 met our inclusion and exclusion criteria. No further papers were found after reviewing the reference lists of our selected studies ^1^^,^^4^^,^^14^^,^^16^, ^17^, ^18^, ^19^, ^20^, ^21^, ^22^, ^23^, ^24^, ^25^, ^26^, ^27^, ^28^, ^29^, ^30^, ^31^, ^32^ (Fig. 1).

### 2.4 Study characteristics

The 20 studies included in our analysis were conducted between 2007 and 2023, with 18 retrospective and 2 prospective studies (Table 1). Further, 15 were conducted in the United States, with two studies being conducted in China, one in India, one in Italy, and one in the United Kingdom. 9 studies were conducted within the ACS-NSQIP database with the remainder being single-institution studies (Table 1). Notably, two studies by Lung et al.^24^^,^^30^ were derived from the same ACS-NSQIP cohort (2007–2020; n = 275,107) but evaluated distinct outcomes and were therefore included separately in the analysis.

**Table 1:** Characteristics of studies included in the analysis.

Study	Design		Sample Size	Study Period	Data Source	Country	Procedure(s)	Pre or Postoperative Hyponatremia	Hyponatremia Cutoff	Minors Score
Naathan et al.^16^	Retrospective cohort		1402 patients (776 TKA and 626 THA)	2019	Single institution	UK	THA, TKA	Both Pre-op and Post-op	Defined as <130, also notes "Treatment was warranted for sodium levels <130 mmol/l or > 145 mmol/l"	18
Wu et al.^17^	Retrospective cohort		395 patients	Jan 2016 to Nov 2018	Single institution	China	THA	Both	Identifies the "threshold for clinical intervention" as Na + level <137 mmol/L	19
Kunze et al.^18^	Retrospective cohort		30,703 patients (15356 TKA and 15347 THA)	2016 to 2019	Single large tertiary academic hospital	USA	THA, TKA	Post-op hyponatremia is the primary variable being investigated/evaluated (but Pre + Post-op incidence values reported for both)	Serum Na <135 mEq/L	20
He et al.^19^	Prospective RCT		94 patients (47 in control group, 47 in experimental group)	April 2019 to January 2020	Single institution	China	TKA	Post-op (∗pre-op Na+ < 135 mmol/L was listed as exclusion criteria)	Not explicitly defined	24
Mukartihal et al.^20^	Retrospective cohort		456 TJA cases reviewed (63 hip replacements, 316 unilateral knee replacements, and 77 bilateral knee replacements)	January 2014 to June 2015	Single institution	India	THA, TKA	Post-op was evaluated (although pre-op was measured as part of the evaluation)	Serum Na less than 135 mmol/L but was further graded as mild (≥130 mmol/L), moderate (≥125–129 mmol/L) and severe (<125 mmol/L)	18
Baker et al.^34^	Retrospective cohort		3071 total TJAs	2015 to 2017	Single high-volume academic center	USA	THA, TKA	Both Pre-op and Post-op values equally considered	Serum Na <135 mEq/L. 4 groups based on preoperative nad postoperative sodium values (pre-post): normonatremic-normonatremic (Group 1), normonatremic-hyponatremic (Group 2), hyponatremic-normonatremic (Group 3), hyponatremic-hyponatremic (Group 4)	22
Sinno et al.^14^	Retrospective cohort		402 patients	2016 to 2017	Single high-volume academic center	Italy	THA, TKA	Post-op	Serum Na <135 mmol/L, hyponatremia could be mild [130 ≥ Na+ (mmol/L) ≤ 134], moderate [125 ≥ Na+ (mmol/L) ≤ 129], severe [Na+ (mmol/L) ≤ 124]	19
Tanenbaum et al.^21^	Retrospective cohort		59,236 THA procedures	2012 to 2014	ACS-NSQIP database	USA	THA	Pre-op	Serum Na<135 mEq/L = Hyponatremia Serum Na 135-145 mEq/L = Normonatremia Serum Na> 145 mEq/L = Hypernatremia	20
Abola et al.^22^	Retrospective cohort		88,103 TKA patients	2012 to 2014	ACS-NSQIP database	USA	TKA	Pre-Op	Serum Na<135 mEq/L = Hyponatremia Serum Na 135-145 mEq/L = Normonatremia Serum Na> 145 mEq/L = Hypernatremia	20
Ondeck et al.^23^	Retrospective cohort		92,093 THA patients	2011 to 2015	ACS-NSQIP database	USA	THA	Pre-op	There were 3 categories for sodium values. The cutoff values were: (abnormally low sodium: <135 mEq/L, normal sodium: 135–145 mEq/L, and abnormally high sodium: >145 mEq/L)	20
Lung et al., 2023a^24^	Retrospective cohort		275107 THA patients	2007 to 2020	ACS-NSQIP database	USA	THA	Pre-op	Normal serum Na: 135-147 mmol/L	20
Tischler et al.^25^	Prospectively maintained database study		3162 patients (3721 TJA procedures)	Jan 2015 to Dec 2017	Single institution	USA	THA, TKA	Post-op is the primary variable being evaluated, Both Pre-op and Post-op measured	Normal serum Na- 135 to 146 mEq/L	20
Halawi et al.^26^	Retrospective observational		351 patients	2015 to 2017	Single institution	USA	THA	Post-op	Normal serum Na: 135-145 mEq/L	18
Wester et al.^4^	Retrospective observational		389,672 procedures (244,538 TKAs + 145134 THAs)	2011 to 2017	ACS-NSQIP database	USA	THA, TKA	Pre-op	Serum Na<136 mEq/L = Hyponatremia Serum Na>145 mEQ/L = Hypernatremia	20
Khoshbin et al.^27^	Retrospective cohort		216501 patients (137,969 TKA + 78,532 THA)	2011 to 2018 (2011-16: Developmental Cohort, 2017 & 18: Validation cohort)	ACS-NSQIP database	USA	THA, TKA	Pre-op	Normal serum Na = 135-147 Hyponatremia<135 mEq/L	20
Kildow et al.^28^	Retrospective observational		767 patients (441 TKA + 323 THA)	Jan 2012 to Sept 2014	Single institution	USA	THA, TKA	Post-op (Preop levels were taken as well)	Normal serum Na = 135-145 mEq/L	18
Lakomkin et al.^29^	Retrospective observational		121,977 patients (76,041 TKA + 45,936 THA)	2006 to 2013	ACS-NSQIP database	USA	THA, TKA	Preop	Normal serum Na = 135-145 mEq/L	19
Lung et al., 2023b^30^	Retrospective cohort		275,107 patients	2007 to 2020	ACS-NSQIP database	USA	THA	Pre-op	Normal serum Na: 135-147 mmol/l	20
Wei et al.^31^	Retrospective cohort		28,742 patients ANN included n = 25,115 due to missing data for the key variables used in the model	2018	ACS-NSQIP database	USA	TKA	Pre-op	NR	20
Urban et al.^32^	Retrospective cohort		78,492 procedures (20109 TKA + 18372 THA)	2009 to 2014	Single orthopedic specialty hospital	USA	THA, TKA	Pre-op	NR	19
										

THA = total hip arthroplasty; TKA = total knee arthroplasty; UK = United Kingdom; USA = United States of America; ACS-NSQIP = American College of Surgeons National Surgical Quality Improvement Program; Na = sodium, mEq/L = milliequivalents per liter; mmol/L = millimoles per liter.

### 2.5 Risk of bias in individual studies

Using the Methodological Index for Nonrandomized Studies (MINORS) tool,^33^ two reviewers (AG and SP) independently evaluated the quality of included studies. This is a validated assessment tool that grades noncomparative studies from 0 to 16 and studies with control groups from 0 to 24 based on criteria related to study design, outcomes assessed, and follow-up. Across these domains, each item is scored 0 if not reported, 1 when reported but inadequate, and 2 when reported and adequate. The mean MINORS score was 19.7 ± 1.4.

### 2.6 Data extraction and analysis

Data extraction from included articles was conducted by two independent researchers (AG, SP). To reach consensus, disagreements were addressed by a third reviewer (JR). The primary study objectives were threefold: prevalence of perioperative hyponatremia, predictors of postoperative hyponatremia, and outcomes associated with perioperative hyponatremia. In addition, we included sample size, study design, country, dataset, study period, hyponatremia definition, and stratification in data collection. Predictors of hyponatremia consisted of metrics including demographics, medication history, and perioperative surgical information. Postoperative complications consisted of metrics including PROMS and functional metrics, mortality, LOS, readmissions, and revision/reoperation. In the analysis, multivariate outcomes were only recorded if they were provided alongside univariate and/or bivariate outcomes.

## 3 Results

### 3.1 Prevalence of perioperative hyponatremia

A total of 17 studies reported perioperative hyponatremia prevalence in TJA (Table 2). Preoperative rates ranged from 2.1% to 17.8%, whereas postoperative rates ranged from 2.3% to 41.3%. Large national cohorts reported narrower preoperative ranges (3.7–5.2%). One study stratified perioperative sodium profiles, reporting 9.4% postoperative hyponatremia with normal preoperative sodium, 2.1% corrected preoperative hyponatremia, and 3.8% persistent hyponatremia.^1^ Mukartihal et al. reported higher rates, including 37.3% preoperative and 84.9% postoperative hyponatremia.^20^ One study found that most postoperative hyponatremia developed within 24 h.^14^

**Table 2:** Prevalence of perioperative hyponatremia in patients undergoing THA or TKA.

Study	Preoperative Rate	Postoperative Rate	Hyponatremia Cutoff
Naathan et al.^16^	52/1402 (3.7%)	123/1402 (8.8%)	<130 mmol/L
Wu et al.^17^	10/395 (2.5%)	9/395 (2.3%)	Threshold for clinical intervention defined as <137 mmol/L
Kunze et al.^18^	5480/30703 (17.8%)	NR	Serum Na <135 mEq/L
Mukartihal et al.^20^	170/456 (37.3%), 143 mild, 18 moderate, 9 severe	84.9% (80% of these were mild, 16% moderate, and 4% severe)	Serum Na <135 mmol/L but further graded as mild (≥130 mmol/L), moderate (≥125–129 mmol/L) and severe (<125 mmol/L)
Baker et al.^34^	5.9%; 2.1% Group 3 (preop hyponatremia, postop normonatremia), 3.8% Group 4 (preop hyponatremia, postop hyponatremia)	13.2%; 9.4% Group 2 (preop normonatremia, postop hyponatremia), 3.8% Group 4 (preop hyponatremia, postop hyponatremia)	Serum Na <135 mEq/L
Tanenbaum et al.^21^	3051 (5.2%)	NR	Serum Na <135 mEq/L
Abola et al.^22^	3763 (4.3%)	NR	Serum Na <135 mEq/L
Ondeck et al.^23^	THA – 4141 (4.50%)	NR	Serum Na <135 mEq/L
Lung et al., 2023a^24^	THA – 12214 (4.8%)	NR	<135 mmol/L
Tischler et al.^25^	3.4% abnormal Na	13.3% hyponatremia, 25.6% abnormal Na. Among patients with abnormal Na, 52.0% (496 of 954) and 48.0% (458 of 954) were hyponatremic or hypernatremic, respectively.	Normal serum Na 135 – 146 mEq/L
Wester et al.^4^	6.9%	NR	Serum Na <136 mEq/L = Hyponatremia Serum Na >145 mEq/L = Hypernatremia
Khoshbin et al.^27^	7981/216501 (3.7%)	NR	Serum Na <135 MmEq/L
Kildow et al.^28^	NR	317 (41.3%) abnormal Na	Normal serum Na 135-145 mEq/L
Lung et al., 2023b^30^	THA – 4.8%	NR	Normal serum Na 135-147 mmol/L
Urban et al.^32^	5447/38481 (14.2%)	NR	NR
Sinno et al.^14^	NR	108 (26.9%), 66.7% of those developed hyponatremia within 24 h postoperatively. Mean postoperative hyponatremia was 133.38 (127.78–134.85) mmol/L.	Serum Na <135 mmol/L, hyponatremia could be mild [130 ≤ Na+ (mmol/L) ≤ 134], moderate [125 ≤ Na+ (mmol/L) ≤ 129], severe [Na+ (mmol/L) ≤ 124]
He et al.^19^	NR	OECNS group (3, 6.4%); control group (10, 21.3%); p = 0.036	NR

THA = total hip arthroplasty; TKA = total knee arthroplasty; Na = sodium; NR = not reported; OECNS = oral electrolyte-carbohydrate nutrition supplement; mEq/L = milliequivalents per liter; mmol/L = millimoles per liter; perioperative rates provided in terms of number of patients.

### 3.2 Predictors of postoperative hyponatremia

Four studies identified abnormal preoperative sodium as the strongest predictor of postoperative hyponatremia, including in a machine-learning model of 30,703 TJA patients^16^^,^^18^^,^^25^^,^^28^ (Table 3). Notably, Naathan et al. reported the highest risk (OR 12.33, 95% CI 7.44–21.51).^16^ Advanced age, diabetes, and bilateral or prolonged procedures were also associated with increased risk.^14^^,^^16^^,^^18^^,^^20^^,^^26^^,^^28^^,^^34^ Thiazide diuretics show differing results with regards to abnormal postoperative sodium levels. In a retrospective cohort study of 402 TJA patients, chronic use of thiazides was associated with a decreased risk of developing postoperative hyponatremia,^14^ while Tischler et al. found that patients taking a thiazide diuretic had 1.35 (95% CI: 1.24-1.46) times higher odds of abnormal postoperative Na.^25^ Angiotensin-converting enzyme (ACE) inhibitors were identified in two studies^20^^,^^25^ to increase risk of developing postoperative hyponatremia {(OR: 1.664, 95% CI: 1.178-2.336)^16^ and (OR: 1.12, p < 0.001) ^25^} (Table 3).

**Table 3:** Predictors of postoperative hyponatremia in patients undergoing THA or TKA.

Study	Predictors (variable – OR)
Baker et al.^34^	Older age (p < 0.001), use of general anesthesia (p < 0.01), higher CCI (p < 0.01), CHF (p = 0.01), and CKD (p < 0.01), among others, are risk factors for postoperative hyponatremia.
Naathan et al.^16^	Risk factors identified were age (OR: 1.035 (95% CI: 1.024 to 1.047), p < 0.001), preoperative abnormal sodium level (OR: 12.33 (95% CI: 7.44 to 21.51), p < 0.001), and long-term use of NSAIDs (OR 0.6786 (95% CI: 0.4997-0.9121)), ARBs (OR 1.664 (95% CI: 1.178-2.336)), and corticosteroids (OR 2.099 (95% CI: 1.002-4.349)).
Kunze et al.^18^	A combination of 6 of the potential 19 predictor variables optimized the accuracy of hyponatremia prediction: preoperative serum sodium concentration, age, intraoperative blood loss, procedure time, BMI, and ASA score. Preoperative serum sodium concentration was the most important factor. Threshold values that were associated with greater hyponatremia risk were a preoperative serum sodium concentration of ≤138 mEq/L, an age of ≥73 years, an ASA score of >2, intraoperative blood loss of >407 mL, a BMI of ≤26 kg/m2, and a procedure time of >111 min.
Mukartihal et al.^20^	Preoperative electrolyte anomaly was a consistent finding (16/16 = 100% in severe hyponatremia and 38/60 = 63% in moderate hyponatremia). Thaizides, ACE inhibitors, and longer surgeries like bilateral TKRs had more cases of hyponatremia. Of the 16 severe postoperative hyponatremic patients, all had a prior electrolyte abnormality identified. Of the 60 moderate hyponatremic patients, 38 had a preoperative electrolyte anomaly. Patients undergoing TKA appeared more prone to experiencing hyponatremia than THA patients. Another interesting observation was that TKA patients had greater total fluid intake (both intra- and postoperatively) compared with the THA, with bilateral TKA being the highest.
Sinno et al.^14^	Chronic use of thiazides was the only variable associated to a decreased risk of developing postoperative hyponatremia (OR: 0.39; p = 0.03). Hemoglobin postoperative values (OR: 1.22; p = 0.03), the need of postoperative transfusion (OR: 2.50; p = 0.02) and diabetes (OR: 2.70; p = 0.01) were associated to an increased risk of postoperative hyponatremia.
Tischler et al.^25^	The odds of an abnormal postoperative Na result were 2.15 times higher (95% CI: 1.82-2.54) for patients with an abnormal preoperative Na result compared to those with normal preoperative Na. Thiazide diuretic use increased the odds by 1.35 (95% CI: 1.24-1.46). Other significant associations included male sex (OR: 1.29), ACE/ARB use (OR: 1.12), bilateral procedures (OR: 1.56), and CCB use (OR: 1.10), all with p < 0.001. Apart from abnormal preoperative Na, other risk factors had small effect sizes on postoperative Na values.
Halawi et al.^26^	For abnormal postop Na levels, there was a significant association with abnormal baseline Na levels (OR: 8.08, p < 0.001), diabetes (OR: 3.14, p = 0.007), and not using tranexamic acid (OR: 3.05, p = 0.001)
Kildow et al.^28^	Multivariable logistic regression analysis found older age was associated with a minimal increased risk of an abnormal postoperative sodium value (OR: 1.02, 95% CI: 1.00-1.04, p = 0.047) and decreased preoperative sodium value (OR: 0.72, 95% CI: 0.67-0.78, p < 0.001).

THA = total hip arthroplasty; TKA = total knee arthroplasty; CCI = Charlson Comorbidity Index; CHF = congestive heart failure; CKD = chronic kidney disease; NSAID = non-steroidal anti-inflammatory drug; BMI = body mass index; ACE = angiotensin-converting enzyme; ARB = angiotensin II receptor blocker; CCB = calcium channel blocker; ASA = American Society of Anesthesiologists; Na = sodium; mEq/L = milliequivalents per liter; OR = odds ratio; CI = confidence interval.

### 3.3 Outcomes associated with preoperative hyponatremia

Nine studies evaluated outcomes associated with preoperative hyponatremia (Table 4). Several reported increased risk of reoperation,^4^^,^^21^^,^^22^ readmission,^4^^,^^23^ and prolonged length of stay,^4^^,^^21^^,^^24^ although some studies found no association with readmission.^21^^,^^22^ Registry data demonstrated increased readmission risk after TKA but not THA.^27^ Similarly, one study found thathigher preoperative sodium was also associated with greater likelihood of same-day discharge.^31^ Individual studies also demonstrated additional complications linked to perioperative hyponatremia included periprosthetic fracture and postoperative delirium.^24^^,^^32^

**Table 4:** Outcomes associated with perioperative hyponatremia in patients undergoing THA or TKA.

	Study	Outcomes
Preoperative Hyponatremia	Tanenbaum et al.^21^	preoperative hyponatremia was significantly associated with 30-day reoperation (OR 1.18; 99% CI: 1.02–1.36) preoperative hyponatremia was significantly associated with increased odds of MMM (odds ratio [OR] 1.17; 99% confidence interval [CI]: 1.03–1.32), MM (OR 1.14; 99% CI: 1.01–1.30), and longer hospital LOS (OR 1.20; 99% CI: 1.13–1.27). preoperative hyponatremia was not significantly associated with greater odds of 30-day readmission (OR 0.91; 99% CI: 0.82–1.01).
	Abola et al.^22^	Preoperative hyponatremia was not significantly associated with different odds of readmission (OR 1.12; 99% CI: 1–1.24) or MM (OR 1.05; 99% CI: 0.93–1.19) compared to normonatremia. However, hyponatremic patients had significantly higher odds of a longer hospital stay (OR 1.15; 99% CI: 1.09–1.21) and reoperation (OR 1.24; 99% CI: 1.05–1.46).
	Ondeck et al.^23^	Patients with preoperative hyponatremia had higher odds of hospital readmission (OR 1.40; CI: 1.22–1.62; p < 0.001). Lower sodium levels were associated with increasing adverse outcomes, peaking at sodium ≤127 mEq/L (32.4% adverse events, 31.2% extended hospital stay; p < 0.001). For extended hospital stay (>3 days), the odds ratio was 1.44 (CI: 1.34–1.56; p < 0.001), compared to 1.27 (CI: 1.05–1.53; p = 0.012) for normal sodium levels.
	Lung et al., 2023a^24^	Multivariate logistic regression model: Low sodium (OR: 1.570, 1.284-1.922, p < 0.001) asso. with periprosthetic fracture {Other associated factors older age, obesity class III, mild-to-moderate eGFR, longer operative time, longer LOS}
	Wester et al.^4^	Preoperative hyponatremia was an independent risk factor for readmission (OR 1.40; 95% CI 1.20-1.63, p < 0.001), increased length of stay (LOS) (OR 1.21; 95% CI 1.13-1.29, p < 0.001), and any 30-day complications (OR 1.26; 95% CI 1.11-1.43, p < 0.001). Within complications, it was associated with higher rates of surgical site infections (SSIs) (OR 1.39; 95% CI 1.03-1.88, p = 0.033) and blood transfusion (OR 1.20; 95% CI 1.07-1.35, p = 0.002). Hyponatremia also increased the risk of reoperation (OR 1.47; 95% CI 1.14-1.89, p < 0.001). Mortality within 30 days had an OR of 1.80 (95% CI 0.96-3.34, p = 0.065).
	Khoshbin et al.^27^	Acute readmission (30-day readmission)--- Multivariable analysis TKA - Sodium was significantly associated with increased rated of readmission when analyzed as continuous variable in both the dev and validation cohort. When clinically relevant cutoffs were applied, preop sodium not associated with increased rate of readmission. Multivariable analysis THA- sodium levels did not prove to significantly influence the odds of acute readmission
	Lung et al., 2023b^30^	1258 (0.5%) experienced a postoperative prosthetic hip dislocation. A greater percentage of dislocation patients had low sodium levels (all patients: 4.8% hyponatremic, dislocation patients: 9.2% hyponatremic, p < 0.001). Multivariate: Preop low sodium {OR: 1.294 (95% CI: 0.921-1.818), p = 0.138} for prosthetic hip dislocation
	Wei et al.^31^	Patients in the same-day discharge cohort were more likely to have higher sodium (p < 0.001, independent-samples *t*-test). Preop sodium remained significant after logistic regression analysis.
	Urban et al.^32^	Preop hyponatremia in POD subgroup- (28.6%) Preop hyponatremia in no POD subgroup- (13.9%) Preop hyponatremia as a risk factor for POD = 2.36 (1.54,3.64) p < 0.001
Postoperative Hyponatremia	Baker et al.^34^	90-day readmission data- Group 1: 6.2%, Group 2: 7.6%, Group 3: 10.6%, Group 4: 18.1% LOS- Group 1: 2.4 days, Group 2: 4.4 days, Group 3: 6.2 days, Group 4: 6.4 days Mortality- Group 4: 2.6% (3 patients), all other groups had 0%. Patients with postoperative hyponatremia (Groups 2 and 4) had greater likelihoods of experiencing any complication and non-home discharge.
	Sinno et al.^14^	Recovery Length- 4.23 days in non-hyponatremia group (A) vs 4.84 days in hyponatremic group (B) (p = 0.77) Type of surgery (TKA vs THA), operation time, number of transfusions, LOS and presence of postoperative symptoms did not show statistically significant differences within groups.
	Halawi et al.^26^	Abnormal lab values did not change the course of care in 338/351 pts (96%). Decision to obtain postop lab test should be based on individual risk factors. Vast majority of electrolyte abnormalities are inconsequential. Abnormal lab values were not associated with 90-day ED visits/readmissions (p = 0.21), abnormal lab values were not associated with increased LOS (p = 0.228).
	Lakomkin et al.^29^	Multivariate logistic regression model for THA: After controlling for all comorbidities and perioperative variables, abnormal sodium was found to be significantly associated with major systemic complications (OR: 1.89; 95% CI: 1.12 to 3.17; p = 0.016). This model showed strong predictive capacity with c-statistic value of 0.77. Multivariate logistic regression model for TKA: No association between sodium and post op complications

THA = total hip arthroplasty; TKA = total knee arthroplasty; ED = emergency department; pts = patients; OR = odds ratio; CI = confidence interval; MM = major morbidity; MMM = major morbidity and mortality.

### 3.4 Outcomes associated with postoperative hyponatremia

Four studies analyzed the association of postoperative hyponatremia with postoperative outcomes (Table 4). Two studies found that postoperative hyponatremia was associated with greater likelihood of major systemic complications.^1^^,^^29^ The first study demonstrated that abnormal postoperative sodium levels were associated with increased odds of major systemic complications in THA patients after adjusting for comorbidities and perioperative factors (OR: 1.89, 95% CI: 1.12–3.17, p = 0.016).^29^ The next study reported that patients in the hyponatremic groups had greater likelihood of experiencing any complication and non-home discharge, with 90-day readmission rates of 7.6% and 18.1% in groups 2 and 4, respectively, compared to 6.2% and 10.6% in groups 1 and 3.^1^ In contrast, Sinno et al. and Halawi et al. found no statistically significant differences in postoperative outcomes between hyponatremic and normonatremic groups.^14^^,^^26^

### 3.5 Management of perioperative hyponatremia and impact of normalization

Three studies assessed strategies for managing hyponatremic patients and impact of serum sodium normalization on outcomes (Table 5). He et al. performed a prospective RCT on 94 patients undergoing TKA over the age of 65 and evaluated efficacy of preoperative oral electrolyte-carbohydrate nutrition supplement (OECNS). They found OECNS increased sodium level and reduced postoperative hyponatremia (6.4% vs 21.3%, *p* = 0.036) with substantial improvements in subjective comfort, electrolytes, and blood glucose without increasing rate of complications.^19^ Baker et al. analyzed patients with postoperative hyponatremia whose sodium returned to within normal limits (normalized) prior to discharge compared with those whose sodium did not normalize. In contrast to He's findings, they found increased length of stay (normalized OR: 5.93; non-normalized OR: 2.88, *p* < 0.001), higher rates of non-home discharge (normalized 46.2%; non-normalized 18.4%, *p* < 0.01), increased 90-day readmission (normalized 11.2%; non-normalized 4.0%, *p* = 0.027), and increases in both medical (normalized 25.0%; non-normalized 3.6%, *p* < 0.001) and surgical complications (normalized 8.8%; non-normalized 0.9%, *p* < 0.001).^1^ Lastly, Tischler et al. reported that 40% of patients with hyponatremia received sodium chloride fluids or tablets; however, only 0.3% of all total joint arthroplasty procedures in the cohort (13 of 3721 cases) had a documented electrolyte-related medicine consultation.^25^

**Table 5:** Management of perioperative hyponatremia and the impact of normalization on outcomes in patients undergoing THA or TKA.

Study	Normalization Protocol	Outcomes
He et al.^19^	Evaluated efficacy of preoperative OECNS on patients aged >65 years undergoing TKA.	Higher sodium level and reduced hyponatremia in intervention group. Higher level of Na+ (OECNS group 140.54 ± 3.39; control group 138.07 ± 5.21; p = 0.008) and reduced rate of hyponatremia (OECNS group 6.4%; control group 21.3%; p = 0.036). OECNS patients experienced generally improved PROMs including preoperative hunger (0.43 ± 0.10), pain (2.30 ± 0.34) and anxiety (9.04 ± 2.71) were significantly lower in OECNS group compared with control group (hunger, 1.19 ± 0.21; pain, 3.79 ± 0.26; anxiety, 11.21 ± 3.02) all p < 0.05 as well as weakness on first postoperative day (OECNS group 3.57 ± 0.24); control group 5.15 ± 0.29; p < 0.001). Length of stay 3.4 for control group, 3.34 for OECNS group
Baker et al.^34^	TJA patients who had postoperative hyponatremia that was normalized to within normal limits prior to discharge were compared to those who did not have their sodium values normalized. No mention of treatment methodology.	LOS (normalized, 5.93 (SD = 5.11); non-normalized, 2.88 (SD = 2.62), p < 0.001), Non-home discharge (normalized, 37 (46.2%); non-normalized, 41 (18.4%), p < 0.01), 90-day readmission (normalized, 9 (11.2%); non-normalized, 9 (4.0%), p = 0.027), any complication (normalized, 22 (27.5%); non-normalized, 10 (4.5%), p < 0.001), medical complication (normalized, 20 (25.0%); non-normalized, 8 (3.6%), p < 0.001), surgical complication (normalized, 7 (8.8%); non-normalized, 2 (0.9%), p < 0.001)
Tischler et al.^25^	Forty percent of hyponatremic patients received NaCl fluid/tablets.	Electrolyte-related medicine consultation rate was 0.3% (13/3721)

THA = total hip arthroplasty; TKA = total knee arthroplasty; OECNS = oral electrolyte-carbohydrate nutrition supplement; NaCl = sodium chloride; LOS = length of stay; SD = standard deviation; OR = odds ratio; CI = confidence interval; PROM = patient recorded outcomes measure.

## 4 Discussion

Hyponatremia is a common perioperative electrolyte abnormality in TJA and is associated with adverse clinical outcomes. This review found that perioperative hyponatremia was frequent and was more consistently associated with complications, length of stay, and reoperation when present preoperatively than postoperatively. These findings suggest that preoperative hyponatremia may reflect underlying physiologic vulnerability rather than a transient postoperative disturbance alone. Clinically, these results support targeted preoperative sodium assessment and risk stratification, particularly in elderly or comorbid patients, rather than indiscriminate postoperative laboratory testing. Incorporating perioperative sodium status into optimization pathways may allow surgeons to better identify high-risk patients, allocate resources efficiently, and refine perioperative care strategies within value-based arthroplasty practice.

### 4.1 Perioperative hyponatremia rates

Perioperative hyponatremia rates varied widely, with broader ranges in single-institution cohorts and narrower ranges in large national databases. Generally, our studies report lower preoperative than postoperative hyponatremia rates with one study finding a lower postoperative rate.^17^ Similarly, Hennrikus et al. found 7% and 30% postoperative hyponatremia out of 1067 orthopaedic surgery patients admitted to a single institution.^7^ The perioperative hyponatremia rates found in TJA align with prior analyses in spinal surgery patients that found a 15.8%^35^ and 28%^36^ postoperative rate in studies of 582 and 200 patients, respectively. These findings are biologically plausible, as major orthopaedic procedures may disrupt fluid and electrolyte homeostasis through nonosmotic stimulation of arginine vasopressin (AVP), triggered by pain, nausea, hypotension, opioid administration, and inflammatory stress.^37^^,^^38^ Hypotonic fluids or excess free water intake can exacerbate dilutional hyponatremia, whereas ERAS pathways (opioid-sparing analgesia, nausea prophylaxis, and goal-directed fluid management) may reduce these perioperative triggers.^39^

### 4.2 Risk factors associated with postoperative hyponatremia

Abnormal preoperative sodium levels, advanced age, diabetes, and bilateral or prolonged procedures were the most consistent predictors of postoperative hyponatremia, with preoperative sodium status consistently emerging as the strongest factor. These findings support selective preoperative sodium assessment in patients with identifiable risk factors for electrolyte disturbance.^40^ Prior work suggests that hyponatremia may represent a marker of physiologic vulnerability rather than an isolated electrolyte disturbance. One study linking hyponatremia with falls, fractures, osteoporosis, and cognitive impairment proposed that is may reflect unhealthy aging.^41^ Consistent with this concept, several medication classes identified as predictors of postoperative hyponatremia are commonly prescribed for chronic cardiometabolic disease and may therefore serve as proxies for underlying comorbidity burden and reduced physiologic reserve. In addition, bilateral procedures and prolonged operative times were also procedural predictors, and thus, strategies aimed at minimizing operative time, limiting blood loss, and optimizing perioperative fluid management may help mitigate these physiologic stressors.^42^ Although thiazide diuretics have been associated with hyponatremia,^43^ findings within TJA populations were discordant across the two included studies.^14^^,^^25^ These differences may reflect variation in patient populations, exposure patterns, and comorbidity burden, and preclude definitive conclusions about their role in postoperative hyponatremia risk.

### 4.3 Outcomes associated with perioperative hyponatremia

In each study that analyzed the association of preoperative hyponatremia with postoperative outcomes, hyponatremia was associated with increased risk of multiple complications including reoperation, readmission, late discharge, and general medical and surgical complications. Outcomes associated with postoperative hyponatremia included major systemic complications rather than surgical complications. However, two of the four studies found no statistically significant differences in outcomes. Therefore, our results imply greater risk associated with preoperative hyponatremia when compared with postoperative hyponatremia. Low sodium levels preoperatively have been proposed to compromise nerve-muscle conduction and balance and decrease general hip bone mineral density scores.^44^ Consequently, in the context of THA bone demineralization has been hypothesized to slow femoral stem osseous integration and increase risk of acute periprosthetic fractures and length of recovery.^45^ On the other hand, postoperative hyponatremia may be more representative of the stresses imposed by TJA rather than underlying poor physiological reserve and chronic health concerns.

#### 4.3.1 Management of perioperative hyponatremia and outcomes associated with normalization

Of the three studies evaluating management of perioperative hyponatremia, methodology and study objectives differed substantially. One study reported that sodium chloride supplementation was commonly used with low complication rates and infrequent need for electrolyte-related consultation, suggesting that NaCl therapy can be safely employed in TJA patients when appropriate dosing guidelines are followed.^25^,^37^ Another study demonstrated that preoperative oral electrolyte-carbohydrate supplementation increased sodium levels and reduced postoperative hyponatremia without increasing complication rates, supporting the safety of preoperative sodium optimization in selected patients.^19^ In contrast, patients whose postoperative hyponatremia normalized prior to discharge had higher complication rates and greater resource utilization, likely reflecting greater underlying medical complexity rather than harm from sodium correction itself.^1^

Hyponatremia has been associated with bone loss, gait disturbances, and demineralization, suggesting that correction prior to surgery may provide physiologic benefit.^40^ Accordingly, some authors have recommended correction even in asymptomatic patients.^24^ However, additional evidence remains limited, and optimal perioperative correction strategies and timing of intervention remain unclear.

The shift toward outpatient and short-stay arthroplasty introduces additional challenges, as most postoperative hyponatremia develops within 24 to 48 h. Routine laboratory testing in all patients is unlikely to be cost-effective, but a risk-stratified approach may be appropriate. Early laboratory evaluation or close follow-up may be reasonable in patients with preoperative hyponatremia, advanced age, diuretic use, bilateral procedures, or substantial comorbidity burden. Prospective studies are needed to define optimal monitoring strategies in contemporary arthroplasty practice.

### 4.4 Limitations

This study has several limitations. First, exclusion of non-English language articles may have resulted in omission of relevant data. Second, substantial clinical and methodological heterogeneity existed among included studies, including variation in definitions of hyponatremia, outcome reporting, and study design, which precluded quantitative meta-analysis. Third, management of perioperative hyponatremia and outcomes associated with sodium normalization remain understudied, with few available studies and considerable variability in treatment strategies and timing of intervention. Residual confounding is possible given the observational nature of most included studies. Finally, although multiple databases were searched, relevant studies indexed elsewhere may have been missed. Despite these limitations, this review summarizes current evidence regarding prevalence, predictors, outcomes, and management of perioperative hyponatremia in total joint arthroplasty.

## 5 Conclusion

Hyponatremia is a clinically relevant and modifiable risk factor in TJA, consistently associated with worse perioperative outcomes. Perioperative hyponatremia is prevalent in TJA patients, with rates consistently higher in the postoperative period, reflecting the unique physiologic stressors of major joint surgery. The most commonly identified risk factors of postoperative hyponatremia included abnormal preoperative sodium levels, advanced age, diabetes, and prolonged or bilateral procedures. These findings underscore the importance of preoperative optimization and vigilant postoperative monitoring, particularly in elderly or comorbid patients. Serum sodium normalization demonstrated safety and improved outcomes when conducted preoperatively, but worse outcomes in patients treated for postoperative hyponatremia prior to discharge. Further prospective research is warranted to define evidence-based management protocols, including randomized controlled trials evaluating preoperative sodium optimization and clarifying optimal treatment strategies across the perioperative continuum.

## Guardian/patient's consent

No consent was needed as no patients or human data were included in this study.

## Study location

This study was performed at Case Western Reserve University School of Medicine, Cleveland, OH.

## Registration

PROSPERO registration of the study protocol: CRD42024503307, 28 January 2024.

## Ethical committee approval

Due to the deidentified and aggregated nature of the patient data, it is considered Health Insurance Portability and Accountability Act (HIPAA) compliant and was deemed exempt from Institutional Board Review approval.

## Credit author statement

AG, SP, LM, JRP, and MSL were involved in conceptualization, methodology, formal analysis, visualization, writing original draft, and writing review and editing. AFK was involved in conceptualization, methodology, project administration, formal analysis, visualization, writing original draft, writing review and editing, and supervision

## Funding

No funding to disclose.

## Declaration of competing interest

AFK reports the following disclosures: paid presenter or speaker (Zimmer Biomet), paid consultant (Zimmer Biomet, Ortho Development, United Ortho), IP royalties (Innomed), and board or committee member (AAOS, AAHKS, and Anterior Hip Foundation). AG, SP, LM, JRP, and MSL have nothing to disclose.

## Appendix 1

The Boolean operators “AND” and “OR” were applied along with keywords and Medical Subject Headings (MeSH) terms in the query: ("Arthroplasty, Replacement, Hip"[Mesh] OR "Arthroplasty, Replacement, Knee"[Mesh] OR "Arthroplasty, Replacement"[Mesh] OR "total joint arthroplasty" OR "total knee arthroplasty" OR "total hip arthroplasty" OR "TJA" OR "THA" OR "TKA") AND (“Hyponatremia” OR “Sodium” OR "Diagnostic Tests, Routine"[Mesh] OR “Metabolic Panel” OR “CMP” OR “BMP” OR “renal function panel” OR “RFP”).
